# Piezoelectric Chitosan Microporous Scaffolds for Ultrasound-Driven Schwann Cell Migration and Enhanced Neurotrophins Production

**DOI:** 10.1021/acsbiomaterials.5c01086

**Published:** 2025-10-14

**Authors:** Marta Bianchini, Francesco Iacoponi, Matteo Battaglini, Gianni Ciofani, Silvestro Micera, Leonardo Ricotti, Eugenio Redolfi Riva, Andrea Cafarelli

**Affiliations:** † The BioRobotics Institute, Scuola Superiore Sant’Anna, Piazza Martiri della Libertà 33, 56127 Pisa, Italy; ‡ Istituto Italiano di Tecnologia, Smart Bio-Interfaces, Viale Rinaldo Piaggio 34, 56025 Pontedera, Italy; § Bertarelli Foundation Chair in Translational Neuroengineering, Centre for Neuroprosthetics and Institute of Bioengineering, School of Engineering, École Polytechnique Fédérale de Lausanne (EPFL), 1007 Lausanne, Switzerland

**Keywords:** Schwann cells, low-intensity pulsed ultrasound, piezoelectric nanoparticles, chitosan, nerve regeneration, cell migration

## Abstract

Peripheral nerve injuries often result in nerve damage that significantly compromises functional recovery. Current treatments have substantial limitations. Engineered nerve guidance conduits emerge as a promising alternative, but their efficacy is limited when bridging large gap injuries. Schwann cells, which are essential for nerve regeneration, require a supportive microenvironment to maintain their regenerative function. Recent advances in tissue engineering focus on combining functional biomaterials and external stimuli, such as electrical stimulation, to achieve nerve guidance conduits that enhance regeneration. This study presents a piezoelectric chitosan scaffold loaded with barium titanate nanoparticles, designed for wireless electrical stimulation of Schwann cells through low-intensity pulsed ultrasound. The scaffold is engineered with an anisotropic pore microstructure to provide biomimicry. Morphological and mechanical characterization confirms that the scaffold exhibits structural properties similar to those of native neural tissue. Using a highly controlled in vitro ultrasound system, we optimize stimulation parameters to maximize cell migration and evaluate neurotrophic factor production. Gene expression analyses reveal the upregulation of cell motility and regeneration pathways. These findings demonstrate that ultrasound-activated chitosan scaffolds hold significant potential as a noninvasive tool for improving nerve regeneration, offering a comprehensive in vitro analysis to facilitate future preclinical and clinical translation.

## Graphical abstract

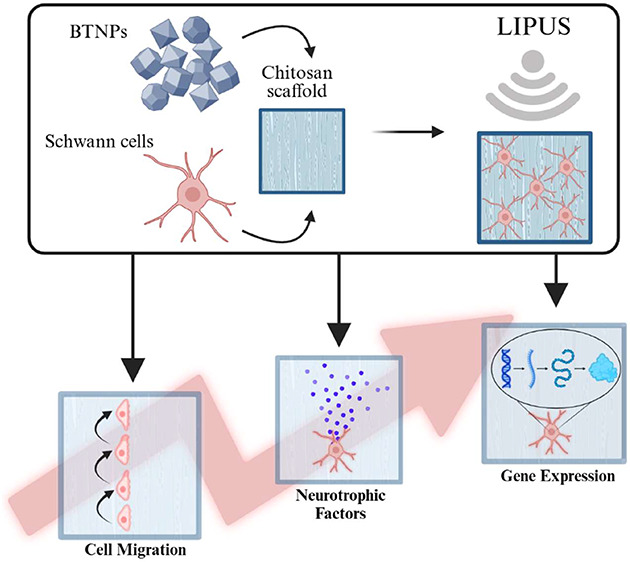

## 1 Introduction

Severe peripheral nerve injuries such as neurotmesis cause a complete loss of axon continuity, which leads to denervation of the target muscle and subsequent functional loss.^1^ Autologous transplantation (autografts) remains the gold standard in clinical practice to restore sensorimotor control in the affected limb. However, its use is constrained by limitations such as limited availability and donor morbidity, which become more significant with the increasing extent of the nerve lesion to be repaired.^2^ Engineered nerve guidance conduits (NGCs) are emerging as promising alternatives to address the limitations of autografts.^3^ However, the effectiveness of NGCs in repairing injuries is still restricted by the extent of the lesion, namely, the nerve gap that needs to be bridged. When this gap exceeds a specific distance −3 cm in humans and 1 cm in rats – known as the limiting gap length (LGL), regenerative efficacy is markedly reduced, particularly in terms of target muscle reinnervation and the resulting functional recovery.^2,4^ Consequently, NGCs have currently been approved for clinical use only in the repair of short-to-midlength nerve gaps.^5,6^

Schwann cells (SCs) play a pivotal role in supporting nerve regeneration processes by migrating along the fibrin cable that reconnects the nerve stumps and guiding the elongation and subsequent myelination of new axonal sprouts across the gap.^7^ However, the efficacy of SCs in supporting his process is time-dependent, with their regenerative phenotype progressively declining within months, leading to reduced neuronal capacity for axonal growth and inefficient regeneration in chronic injuries.^8,9^ In cases of long nerve gaps requiring the regeneration of a significant nerve segment between the injury site and the target muscles, as in brachial plexus injuries, functional recovery of the affected limb is unsuccessful, leading to long-term disabilities and decreased quality of life.^10,11^ The Gordon group has extensively described this phenomenon through experimental models of prolonged muscle denervation in rodents. Their studies demonstrated that denervation exceeding six months leads to a 90% reduction in functional motor units. This process is driven by the progressive inactivation of distal SCs, failure of basal lamina renewal (impossible without SCs and regenerated axons making contact), and fibrosis-induced obstruction of endoneurial tubes, ultimately impairing axonal regeneration and muscle reinnervation.^12^ Therefore, preserving the regenerative phenotype of SCs, facilitating their migration across the nerve gap, and maintaining a supportive environment for reinnervation are critical factors in supporting the regeneration of long-gap lesions.^13^

Recently, new tissue engineering strategies have been explored to improve nerve regeneration. Several studies focused on electrical stimulation, showing that SCs and neurons increase the production of neurotrophic factors, such as nerve growth factor (NGF) and brain-derived neurotrophic factor (BDNF), through a calcium-dependent mechanism.^14–17^ However, these methods require electrode and wire implantations, limiting the stimulation to the proximal stump, increasing the surgical burden, and posing long-term biocompatibility issues. Wireless stimulation strategies are emerging as powerful translational tools, especially those leveraging nanotransducers to precisely convert external energy into targeted stimuli at the cellular level, enabling advanced therapeutic interventions.^18–21^ Piezoelectric nanomaterials such as zinc oxide (ZnO), boron nitride (BN), potassium–sodium niobate (KNN), barium titanate (BaTiO_3_), and poly­(vinylidene fluoride) (PVDF) can generate an electrical signal under mechanical stimulation. When mechanical deformation occurs, the lattice structure shifts from a symmetric to an asymmetric configuration, leading to spontaneous electric polarization.^21,22^ Ultrasound (US) has been studied as a mechanical stimulus to activate the piezoelectricity wirelessly. Several studies have shown that US-activated piezoelectric materials, internalized within the cell cytoplasm or close to the plasma membrane, can increase calcium ion (Ca^2+^) flux in neural cells, induce differentiation of neuroblastoma cells, and enhance neurite outgrowth.^23–25^

Low-intensity pulsed US (LIPUS) is a US therapeutic regime that operates at tuned frequencies above 20 kHz, with an intensity below 3000 mW cm^–2^ and a pulsed pattern designed to limit heat generation. This technology has recently gained significant attention for its potential to enhance tissue repair and promote regeneration, receiving FDA approval for specific applications.^26,27^ Although various hypotheses have been proposed to explain its effects on tissues and cells, many underlying cellular mechanisms remain unclear and are the focus of ongoing research.^28^ Recent studies have explored the combined effect of US and piezoelectric-material-based nerve guidance conduits (NGCs). Xu et al. developed an in vivo-tested NGC incorporating BTNPs and electrospun PVDF nanofibers within a UV-cured hydrogel, further loaded with NGF to assess the synergistic action of neurotrophic release and US-induced piezoelectricity. However, this approach was validated in vitro using PC12 neuronal cells, which differ markedly from SCs both phenotypically and genotypically.^29^ Additionally, the presence of encapsulated NGF within the NGC hinders the precise assessment of the piezoelectric effect relative to the chemical cue in terms of regenerative performance. Other relevant studies reported enhanced regeneration of the injured spinal cord via a US-activated piezoelectric effect using hydrogels incorporating piezoelectric nanomaterials.^30,31^ However, these works focus on a different anatomical district concerning the peripheral nerve, with in vitro studies predominantly focused on assessing neural stem cell differentiation.

Despite recent reports, a comprehensive in vitro analysis of US stimulation protocols to achieve a reproducible and reliable piezoelectric effect on cells remains lacking, hampering the clinical translation of this technique. In this regard, no studies have yet specifically explored the effects of US-assisted piezoelectric stimulation on SCs, which are the key mediators of nerve regeneration.

This study aims to develop a chitosan porous scaffold loaded with barium titanate nanoparticles (Chit.@BTNPs), investigating the effects of LIPUS-assisted piezoelectric stimulation on the behavior of SCs in vitro. First, the morphology and mechanical properties of Chit.@BTNPs were characterized. A custom LIPUS stimulation device, enabling precise tuning of US wave parameters, was used to deliver controlled doses to SCs seeded on the scaffold, allowing the study of their response to different stimulation protocols.^32^ Furthermore, the expression levels of β-NGF, BDNF, and glial cell-derived neurotrophic factor (GDNF) were analyzed during LIPUS stimulation, as these markers are key indicators of specific biological processes, such as inflammation, cellular proliferation, differentiation, and regeneration. Finally, we assessed the modulation of specific gene expression pathways to investigate the biological effects of the LIPUS-assisted piezoelectric stimulation.

## 2 Experimental Section

### 2.1 Fabrication of the Scaffolds (Chitosan and Chit.@BTNPs)

A 3.5% (w/v) chitosan (Heppe Medical Chitosan GmbH, 23704) solution was prepared by dissolving the polymer in deionized (DI) water containing 2% (v/v) acetic acid (Sigma-Aldrich, A6283) under magnetic stirring for 2 h at 45 °C. BTNPs (nominal diameter: ∼60 nm, PlasmaChem GmbH, Berlin, Germany) with different concentrations (0, 0.4, and 1 mg mL^–1^) were dispersed in a solution of DI water and 2% (v/v) acetic acid and sonicated to avoid aggregation of nanoparticles. Later, the 3.5% (w/v) chitosan was added to the solution and stirred for 2 h at 45 °C. Both solutions were filtered with a Spectra Mesh Woven Filter (metallic mesh, 300 μm pore diameter, Spectrum Laboratories, 145908) and degassed with an ultrasonic cleaner (Branson Ultrasonics, Danbury, USA) to remove air bubbles. Chitosan solution with or without BTNPs was injected into a custom-made Teflon mold, shown in Figure 1, allowing the manufacture of four rectangular scaffolds with a 2 mm thickness, a 30 mm length, and a 10 mm width.

**Figure 1:**
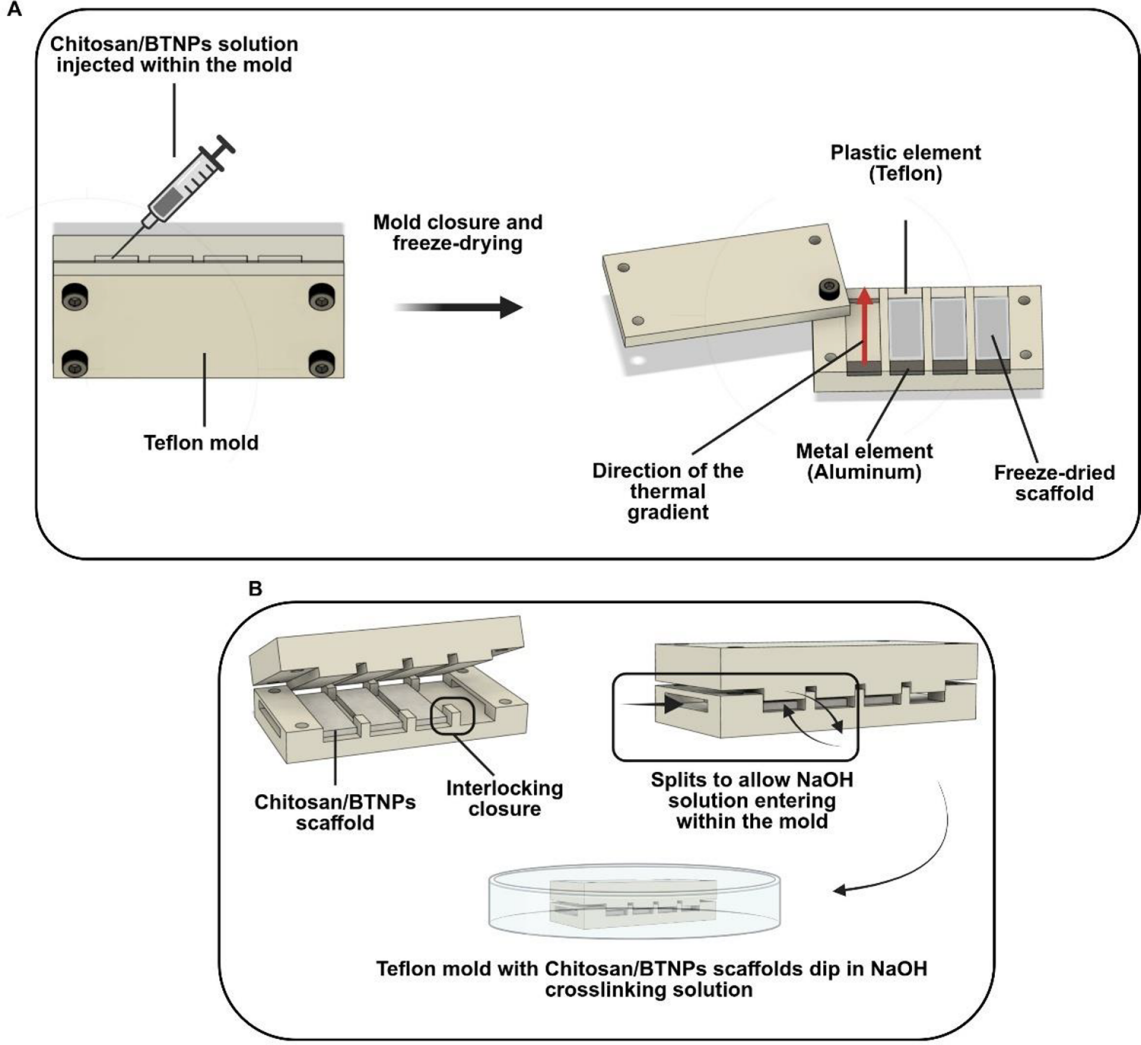
(A) CAD of the custom-made Teflon mold used to manufacture the scaffold. (B) CAD of the Teflon mold used to cross-link the polymeric matrix with a NaOH solution (1% w/v).

The system was engineered to precisely control the scaffold dimensions and porosity during the freeze-drying process. Chitosan solution is injected into a custom-designed mold and sealed at both ends using a Teflon and an aluminum element, respectively (Figure 1A). This configuration allows the establishment of a longitudinal thermal gradient throughout the scaffold during the freezing procedure. After chitosan solution injection, the mold was placed in a refrigerator at *T* = −80 °C overnight and lyophilized with a freeze-dryer (Labconco, Kansas City, USA) for 12 h. To neutralize the remaining acetic acid and cross-link the polymeric chains, freeze-dried scaffolds were placed in a different custom-made Teflon mold with lateral splits (Figure 1B) and immersed in a 1% (w/v) sodium hydroxide solution (NaOH, Sigma-Aldrich, S5881) for 3 h. After this time, the scaffolds were washed with DI water for 15 min and then placed in a 1× phosphate-buffered solution (PBS, Sigma-Aldrich, P4417) overnight to neutralize the pH. A schematic of the scaffold fabrication process is reported in Figure 2. To sterilize the scaffolds for cell experiments, they were placed overnight in a 70% (v/v) ethanol (EtOH) solution. For in vitro tests, each rectangular scaffold was cut manually to obtain a circular structure with a diameter of 1 cm.

**Figure 2:**
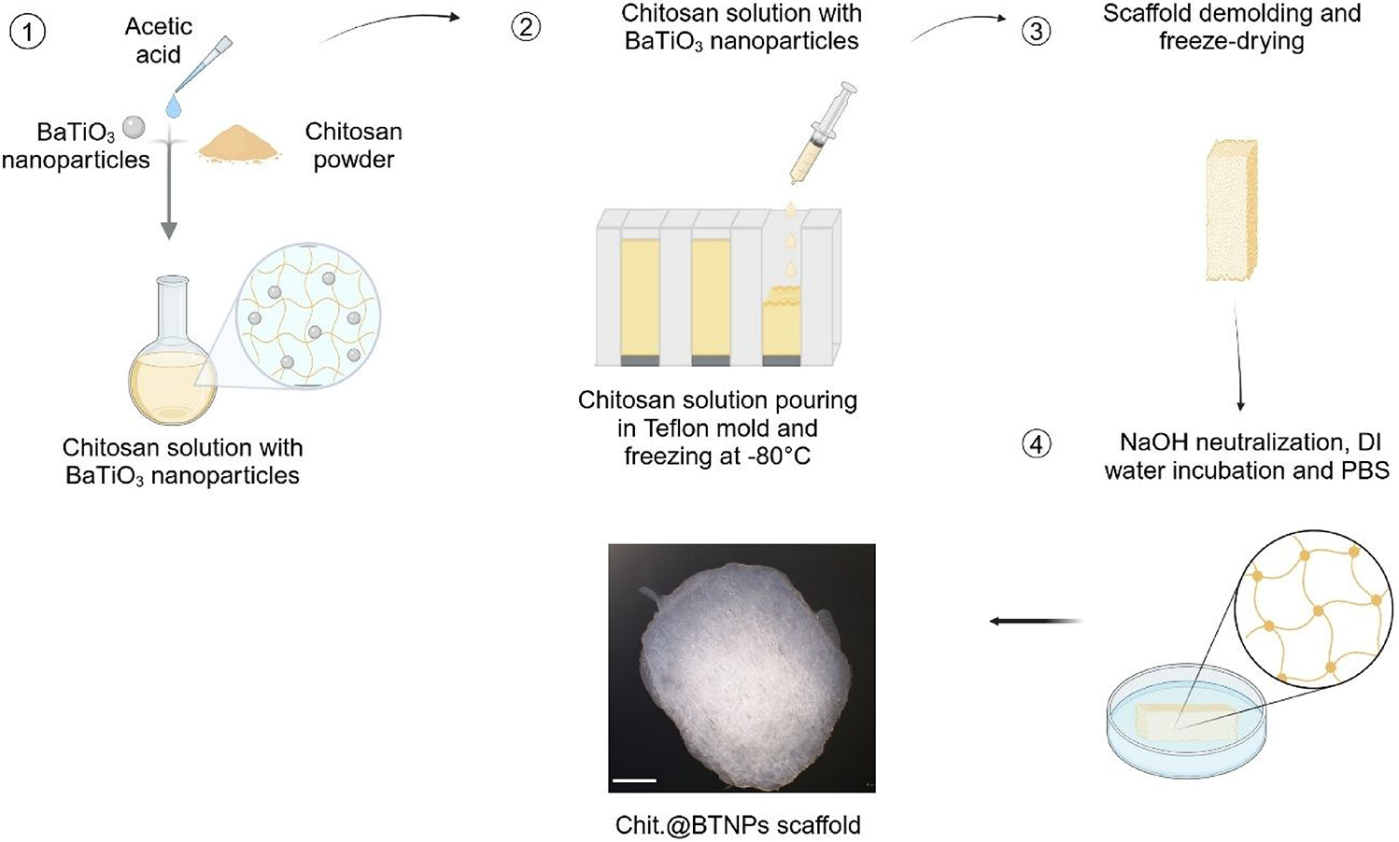
Fabrication process of the Chit.@BTNPs (1 mg mL^–1^) scaffold. Scale bar: 2 mm. Created with permission from Biorender.com.

### 2.2 Zeta Potential Analysis

BTNPs with different concentrations (0.4, 1, and 5 mg mL^–1^) were dispersed in DI water and 2% (v/v) acetic acid. For each sample, three measurements were performed. The measurement parameters were set as follows: medium viscosity 0.8872 cP, temperature 25 °C, and refractive index 1.33. Zeta potential analysis was performed with a Zetasizer Nano ZS90 (Malvern Instruments Ltd., United Kingdom).

### 2.3 Scaffolds Characterization

Morphological characterization of the scaffolds (chitosan and Chit.@BTNPs at concentrations of 0.4 and 1 mg mL^–1^) was performed with scanning electron microscopy (SEM) (Phenom XL, Eindhoven, The Netherlands), equipped with a Bruker Quantax 200 energy dispersive X-ray (EDX) detector (Bruker Nano GmbH, Berlin, Germany) and optical microscopy (HRX01, Hirox, Tokyo, Japan). Before analysis, each sample was washed in DI water and freeze-dried overnight. Before SEM imaging, a thin layer of platinum was sputtered onto the samples, which were further fixed on metal sample holders using carbon tape. To acquire SEM images, two different modes of SEM were used: backscattered electron mode with a voltage of 5 kV, and secondary emission electron mode with a voltage of 15 kV. For EDX spectra acquisition, a beam voltage of 15 kV and map or image mode were set. Pore size was analyzed with ImageJ software (ImageJ, USA^33^) using the Analyze Particle command to calculate the major and minor axis, their ratio, and angular orientation. For each scaffold type, 5 different samples were analyzed. For each sample, 5 images were analyzed, and for each image, 82 ellipses were considered for a total of 410 ellipses. Graphs were generated using GraphPad Prism 9 (GraphPad Software Inc.). Figure S1 illustrates the analysis process for morphological characterization.

Mechanical characterization of scaffolds (chitosan and Chit.@BTNPs at concentrations of 0.4 and 1 mg mL^–1^) was performed in the wet state and after overnight incubation in 1× PBS. Tensile tests were performed by using a tensile machine (Instron, Norwood, MA, USA). Tests were conducted by fixing the scaffolds on the lower and upper grips with a 10 mm gap at a tension rate of 5 mm min^–1^. All data were analyzed with MATLAB R2023b (Mathworks, USA). Five samples from each group were analyzed. Graphs were generated using GraphPad Prism 9 (GraphPad Software Inc.).

### 2.4 Cell Culture

Rat cell line RSC96 (ATCC, CRL-2765) was used as a model of Schwann cells and seeded on the scaffolds at a density of 200,000 cells per sample. Before the seeding, a 1 mm-thick sterile adhesive tape was applied to the center of the scaffolds to create an empty vertical area for the assessment of cell migration. Samples were incubated at 37 °C and 5% CO_2_ and kept in a growth medium (GM) composed of Dulbecco’s Modified Eagle Medium (DMEM, Corning, 10–013-CV), supplemented with 10% fetal bovine serum (v/v, FBS, Sigma-Aldrich, F4135) and 100 IU mL^–1^ penicillin, 100 μg mL^–1^ streptomycin (P/S, Sigma-Aldrich, P4333). Twenty-four hours after seeding, cells were subjected to LIPUS stimulation.

### 2.5 LIPUS Stimulation Setup

A custom-built system was employed to carry out precisely controlled LIPUS stimulations.^32^ Three piezoceramic transducers of identical type (15 mm diameter, 4 MHz central frequency (*f*) for stimulation at 5 MHz) (Precision Acoustics, Dorchester, Dorset, UK) had been previously evaluated in terms of pressure field distributions and intensity calibration, enabling the simultaneous stimulation of three biological samples. The transducers were powered by a 4-channel (2W/channel) signal generator (Image Guided Therapy, Pessac, France), with specialized software for adjusting spatial average-pulse average intensity (*I*_SAPA_), Duty Cycle (DC), pulse repetition *f* (PRF), and stimulation time (*T*). The biological samples (10 × 10 × 2 mm – *L* × *H* × *W*) were housed in a retention system during LIPUS stimulation.^34^ This system was designed to avoid unwanted acoustic reflections and attenuations by utilizing thin membranes (38 μm thick polyurethane film, Stretchlon200, Airtech International Inc.) placed along the acoustic pathway. Additionally, the retention system maintained the sterility of the biological samples by sealing them from external contaminants as it was immersed in DI and degassed water throughout the stimulation process. The acoustic pressure field generated by the transducer was previously characterized through hydrophone mapping and further validated by numerical simulations using the k-Wave MATLAB toolbox, as described in Fontana et al.^32^ This combined characterization confirmed the uniformity of the pressure distribution within the stimulation wells and allowed us to estimate the spatially averaged acoustic intensity within the scaffold volume. Samples were placed so that the ultrasound beam was incident perpendicular to the largest scaffold surface, ensuring homogeneous exposure. Considering the acoustic similarity between chitosan and water, attenuation across the 2 mm scaffold thickness was regarded as negligible.

### 2.6 Design of the LIPUS Stimulation Experiments

The following experimental groups were established: cells were seeded either on plain chitosan or Chit.@BTNPs scaffold, and both of these types of scaffolds were left untreated or stimulated with LIPUS at a *I*_SAPA_ of 250, 500, and 1000 mW cm^–2^ for a total of eight experimental conditions (groups named: “- NPs – LIPUS”, “- NPs + LIPUS_250”, “- NPs + LIPUS_500”, “- NPs + LIPUS_1000”, “+ NPs – LIPUS”, “+ NPs + LIPUS_250”, “+ NPs + LIPUS_500”, and “+ NPs + LIPUS_1000”). The *f*, DC, PRF, and *T* were set to 5 MHz, 20%, 1 kHz, and 5 min per day for a total of three consecutive days, respectively. Such parameters were chosen based on a previous study in which an increment of neurotrophic factor secretion was observed in RSC96 after LIPUS stimulation.^35^ All analyses were performed 24 h after completion of the 3-day stimulation protocol, which therefore represents the final experimental time point.

### 2.7 Cell Viability and Migration Assay

Qualitative analysis of cell viability and cell migration was assessed using a LIVE/DEAD viability/cytotoxicity assay (Invitrogen, L3224). Briefly, the GM was removed and replaced with 1× PBS containing 2 μM calcein-AM and 4 μM ethidium homodimer-1 (EthD-1). After incubation at room temperature for 30 min, scaffolds were washed with 1X PBS and observed under a confocal laser scanning microscopy system with NISElements software (Nikon, Amsterdam, The Netherlands) to acquire two-dimensional confocal images. Four samples from each group were analyzed. For the calculation of the percentage of green pixels in the region of interest, three different areas were analyzed for each image using the appropriate MATLAB R2023b code.

### 2.8 Cell Proliferation Assay

Scaffolds were analyzed using a Quant-iT PicoGreen dsDNA Assay Kit (Invitrogen, p11496). Briefly, GM was removed, cells were lysed in 500 μL of DI water, and aliquots of 50 μL were transferred to a 96-well black round-bottom polystyrene microplate (Corning, 3792), prepared according to the manufacturer’s instructions. After 10 min of incubation in the dark at room temperature, the fluorescence intensity was read with the VICTOR Nivo Multimode plate reader (HH3500, PerkinElmer, Waltham, MA, USA), with an excitation wavelength of 485 nm and an emission wavelength of 535 nm. Results were converted to numeric values by using standard curves. Three samples from each group were analyzed.

### 2.9 Metabolic Activity Assay

Scaffold cultures were analyzed using PrestoBlue Cell Viability Reagent (Invitrogen, A13262). Briefly, the GM was removed and replaced with DMEM containing 10% (v/v) of the reagent described above. After the solution was incubated in a 96-well black round-bottom polystyrene microplate at 37 °C for 60 min, the VICTOR Nivo Multimode plate reader was used to read the fluorescence signal, with an excitation wavelength of 560 nm and an emission wavelength of 590 nm. All of the values were normalized to the respective dsDNA concentration. Three samples from each group were analyzed.

### 2.10 Cytokine Release Quantification

Supernatants were collected, and neurotrophic cytokine production was analyzed through the Rat β-NGF ELISA kit (Thermo Scientific, ERNGF), Rat BDNF ELISA Kit (Thermo Scientific, ERBDNF), and Rat GDNF ELISA Kit (Thermo Scientific, ERA19RB) following the manufacturer’s instructions. The absorbance signal was read with a VICTOR Nivo Multilabel plate reader, setting a primary wavelength of 450 nm for all of the kits. Results were converted to numeric values using standard curves. All protein concentration values were normalized to the respective dsDNA concentration. Three samples from each group were analyzed.

### 2.11 RT2 Profiler PCR Array

Gene expression analysis was performed with RT2 Profiler PCR Arrays (Rotor-Disc 100 format) for Rat Cell Motility (QIAGEN, 330231 PARN-128 ZR), which interrogated 84 genes specifically involved in cytoskeletal remodeling, adhesion, extracellular matrix remodeling, and migration-related signaling. This panel was deliberately selected to evaluate whether piezoelectric stimulation modulates Schwann cell motility, which is identified as the key biological bottleneck in peripheral nerve regeneration. A quantity of 800 ng of total RNA was used to generate cDNA using the RT2 First Strand Kit; Real-time qRT-PCR was performed with the Rotor-Gene Q System (QIAGEN, Germantown, MD, USA). Data analysis was carried out with QIAGEN’s Gene Globe Data Analysis Center using a software-based tool. The geometric mean of 5 housekeeping/reference genes (ACTB, B2M, HPRT1, LDHA, and RPLP1) was used to normalize the raw data. The data analysis web portal calculated fold change/regulation using the ΔΔ*C*(*T*) method.^36^

### 2.12 Statistical Analysis

Data were normally distributed (Shapiro-Wilk test with a significance threshold set at *p* = 0.05) and presented as mean ± std with bar plots. One-way ANOVA with Dunnett’s multiple comparisons test was used to identify statistically significant differences between experimental groups in the morphological characterization of scaffolds. Statistical differences in the mechanical characterization were assessed using One-Way ANOVA with Tukey’s multiple comparisons test. One-way ANOVA with Tukey’s multiple comparisons test was used to identify statistically significant differences, and then in cell migration, metabolic activity, and cytokine release assessments. Student's *t*-test was used to assess differences in gene expression analysis. The results were considered statistically different for *p* < 0.05. Statistical differences were defined as * = *p* < 0.05, ** = *p* < 0.01, *** = *p* < 0.001, and **** = *p* < 0.0001. All analyses were performed using GraphPad Prism 9. Unless otherwise specified, all reported N values refer to biological replicates.

## 3 Results

### 3.1 Piezoelectric Scaffold Characterization

The colloidal stability of BTNPs dispersed in an aqueous solution containing 2% (v/v) acetic acid at different concentrations (0.4, 1, and 5 mg mL^–1^) was assessed by measuring their Zeta Potential. This solution was chosen as it represents the solvent used to prepare the chitosan solution for further scaffold fabrication. The analysis revealed that the Zeta Potential for concentrations up to 1 mg mL^–1^ was approximately 32.8 ± 5.6 mV, whereas, for a concentration of 5 mg mL^–1^, it decreased to approximately 16.7 ± 43.8 mV, as shown in Table 1 and in Figure S2.

**Table 1:** Zeta Potential (Expressed as Mean ± Standard Deviation) of BTNPs Dispersed With Different Concentrations

BTNPs concentration [mg mL^–1^]	zeta potential [mV]
0.4	46.6 ± 6.2
1	32.8 ± 5.6
5	16.7 ± 43.8

These results demonstrated that BTNP dispersion with a concentration of up to 1 mg mL^–1^ exhibited good colloidal stability, as particles with Zeta Potential exceeding ± 30 mV are generally considered stable.^37^ Colloidal stability indicates the effective dispersion of solid nanoparticles within the liquid phase, allowing for a homogeneous distribution while minimizing flocculation and aggregation phenomena.^38^ For this reason, BTNP concentrations up to 1 mg mL^–1^ were selected for Chit.@BTNPs scaffold fabrication and subsequent morphological and mechanical analyses, as a higher concentration of BTNPs is desirable to enhance the piezoelectric effect.

BTNPs were successfully embedded within the chitosan matrix, as shown by Ba peaks evidenced in the EDX analysis (Figure S3). Scaffolds’ morphology is shown in Figure 3.

**Figure 3:**
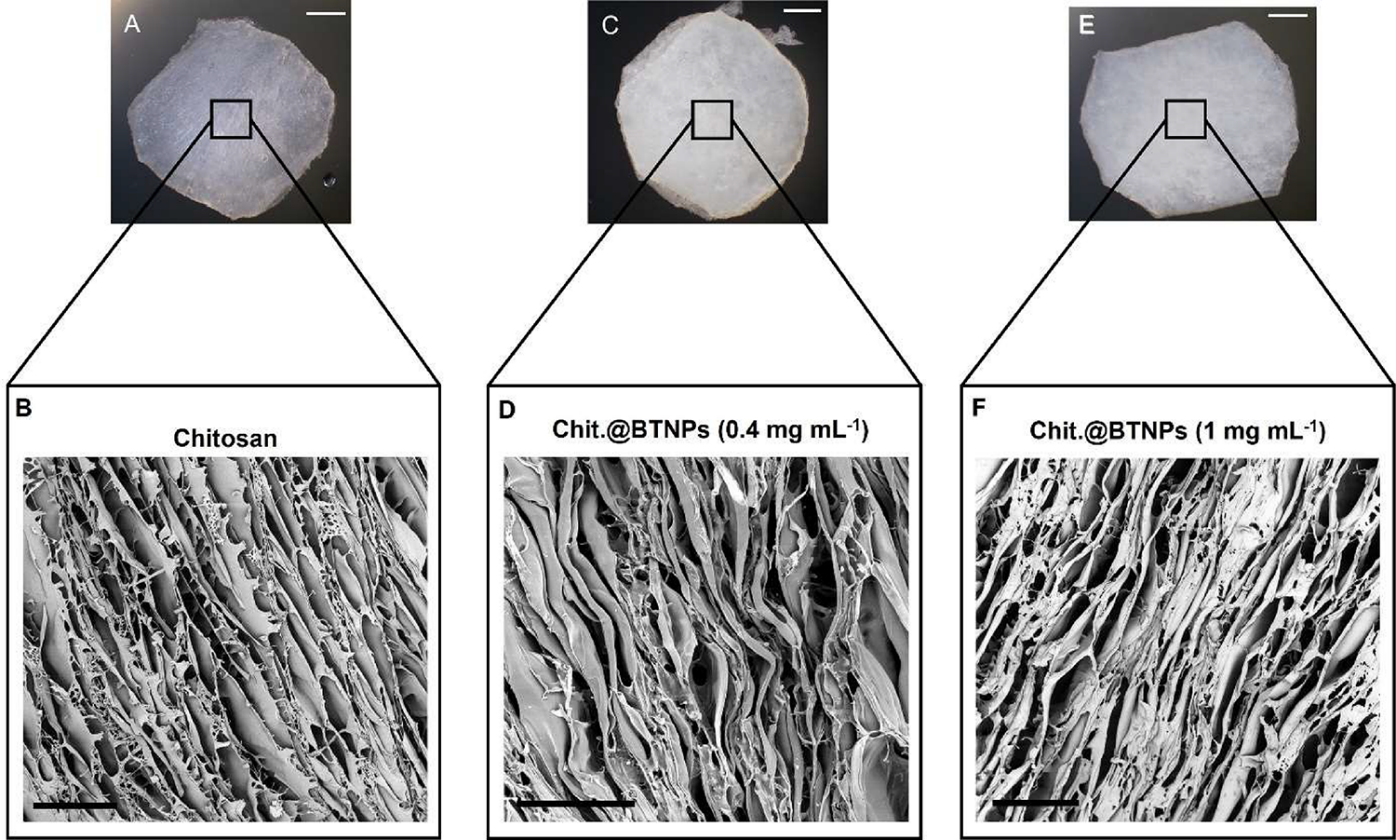
Images of plain chitosan and Chit.@BTNPs scaffolds. (A) Optical microscope and (B) SEM image of a plain chitosan scaffold. (C) Optical microscope and (D) SEM images of Chit.@BTNPs scaffolds loaded with 0.4 mg mL^–1^. (E) Optical microscope and (F) SEM image of Chit.@BTNPs scaffolds loaded with 1 mg mL^–1^ BTNPs concentrations. Scale bars of optical microscopy and SEM images are 2 mm and 300 μm, respectively.

BTNP loading was also confirmed by visual inspection of the scaffolds, corroborated by optical microscopy, which showed that Chit.@BTNPs samples appeared less transparent and exhibited a greater tendency to scatter photons compared with plain chitosan samples (Figure 3A, C, E). Our fabrication process, implemented by the custom-made mold, allowed precise control over the scaffold‘s porous morphology, resulting in samples with highly anisotropic porosity, as shown by SEM images (Figure 3B, D, F). Pore morphology exhibited a pseudoelliptical shape, whose dimensions and orientation can be tailored by varying freezing temperature and the features of the custom-made mold. By tuning these parameters, it is possible to control the nucleation rate and direction of ice crystals in the polymer solution during the freezing process, thus influencing the pores’ microstructure as previously shown by our group.^39–41^ Setting the freezing temperature to −80 °C and securing the polymeric solution with Teflon and aluminum elements, respectively, enabled the formation of lamellar-shaped pores both for Chit.@BTNPs and plain chitosan samples, which resemble the connective architecture of the peripheral nerve.^42,43^

To evaluate the effect of incorporating different BTNP concentrations within the scaffold, pore directionality and morphological analyses were performed upon SEM imaging (Figure 4).

**Figure 4:**
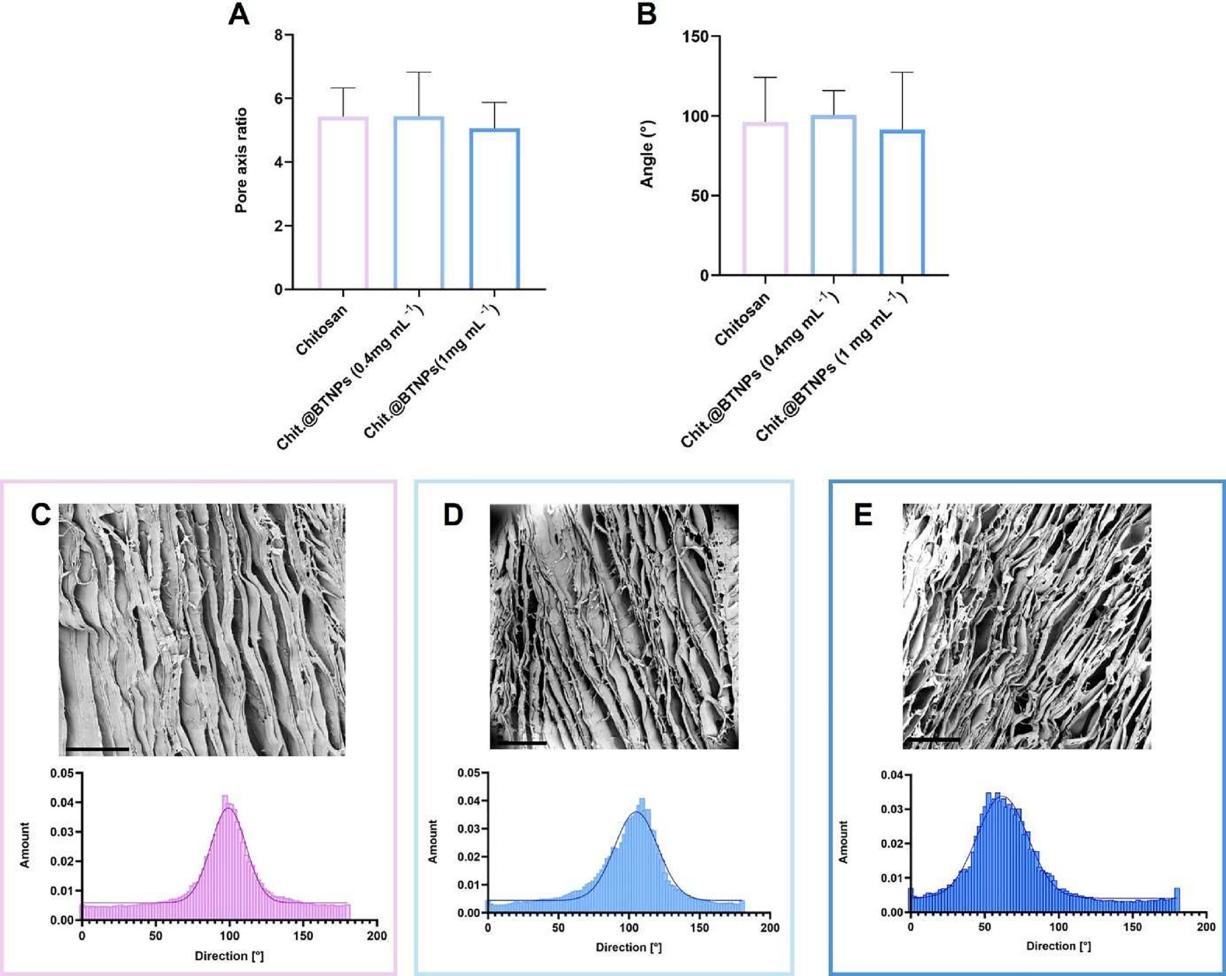
Morphological analysis of the scaffold’s porosity. (A) Pore axis ratio between the major and minor axis of pores on plain chitosan (pink), Chit.@BTNPs (0.4 mg mL^**–**1^, light blue) and Chit.@BTNPs (1 mg mL^–1^, dark blue) scaffolds. (B) Angular orientation of pores on plain chitosan (pink), Chit.@BTNPs (0.4 mg mL^–1^, light blue) and Chit.@BTNPs (1 mg mL^–1^, dark blue) scaffolds. (C) SEM image and corresponding directionality histogram of the chitosan scaffold. (D) SEM image and corresponding directionality histogram of the Chit.@BTNPs (0.4 mg mL^–1^) scaffold. (E) SEM image and corresponding directionality histogram of the Chit.@BTNPs (1 mg mL^–1^) scaffold. Scale bar of the SEM images: 300 μm.

Pores were approximated to elliptical geometries, and the pore axis ratio (aspect ratio), expressed as the ratio between the major and minor axes, was calculated (Figure S1). Morphological analysis indicated that the aspect ratio was 5.43 ± 0.90 for plain chitosan scaffolds and 5.44 ± 1.39 and 5.06 ± 0.82 for Chit.@BTNPs (0.4 mg mL^–1^) and Chit.@BTNPs (1 mg mL^–1^) scaffolds, respectively, confirming an elliptical arrangement (Figure 4A). These findings are consistent with previous studies showing that pores with widths between 20 and 60 μm effectively support axonal growth and cell adhesion.^44,45^ Simultaneously, the angular orientation of pores was calculated, and the results showed a mean value of 96.11° ± 28.20°, 100.50° ± 15.35°, and 91.40° ± 36.12° for plain chitosan, Chit.@BTNPs (0.4 mg mL^–1^) and Chit.@BTNPs (1 mg mL^–1^) scaffolds, respectively, exhibiting a tendency for anisotropic pore disposition endowed through our fabrication process (Figure 4B). This preferential angular arrangement is also reflected in the frequency histograms reported in Figure 4C–E. Interestingly, no statistically significant differences were noted between plain chitosan and Chit.@BTNPs scaffolds, demonstrating that BTNPs loading at different concentrations does not perturb pore morphology. The mechanical properties of the scaffolds were also evaluated (Figure 5).

**Figure 5:**
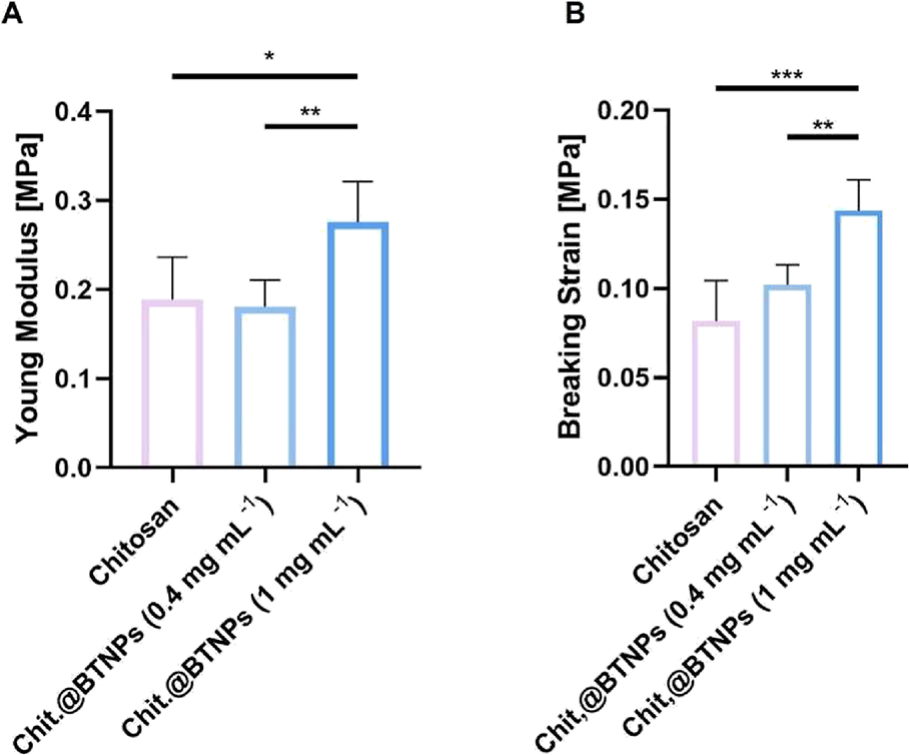
Mechanical Characterization of the scaffold. (A) Tensile Young modulus of plain Chitosan, Chit.@BTNPs (0.4 mg mL^–1^) and Chit.@BTNPs (1 mg mL^–1^) scaffolds. (* = *p* < 0.05, ** = *p* < 0.01 and ***= *p* < 0.001). (B) Breaking strain of plain Chitosan, Chit.@BTNPs (0.4 mg mL^–1^) and Chit.@BTNPs (1 mg mL^–1^) scaffolds.

The stress–strain curves showed a linear relationship between the applied load and deformation. All sample groups withstood deformation up to approximately 50% before reaching their breaking load (Figure S4). Plain chitosan scaffolds exhibited a mean Young’s modulus of 0.19 ± 0.05 MPa, whereas Chit.@BTNPs (0.4 mg mL^–1^) and Chit.@BTNPs (1 mg mL^–1^) scaffolds showed a mean Young’s modulus of 0.18 ± 0.03 and 0.27 ± 0.04 MPa, respectively (Figure 5A). Chit.@BTNPs (1 mg mL^–1^) samples exhibited a significantly higher Young’s modulus and breaking strain (Figure 5B) compared to the other groups, indicating that the loading of different concentrations of BTNPs influences the stiffness of the scaffold. Nonetheless, these results are consistent with the reported Young’s modulus of the native nerve, which is approximately 0.5 MPa.^46^ Overall, these results demonstrate that the fabrication process allowed us to achieve chitosan scaffolds with a highly anisotropic porous microstructure that remained stable across different BTNP concentrations. Additionally, all scaffolds exhibited good tensile strength and stiffness comparable to those of the peripheral nerve ones. Based on these findings and with the idea of maximizing the piezoelectric effect, Chit.@BTNPs (1 mg mL^–1^) scaffolds were selected for further in vitro investigations.

### 3.2 Cell Viability and Migration Assay

Viability tests indicated excellent biocompatibility of the scaffolds with SCs, even with BTNPs incorporated into the chitosan matrix (Figure 6A).

**Figure 6:**
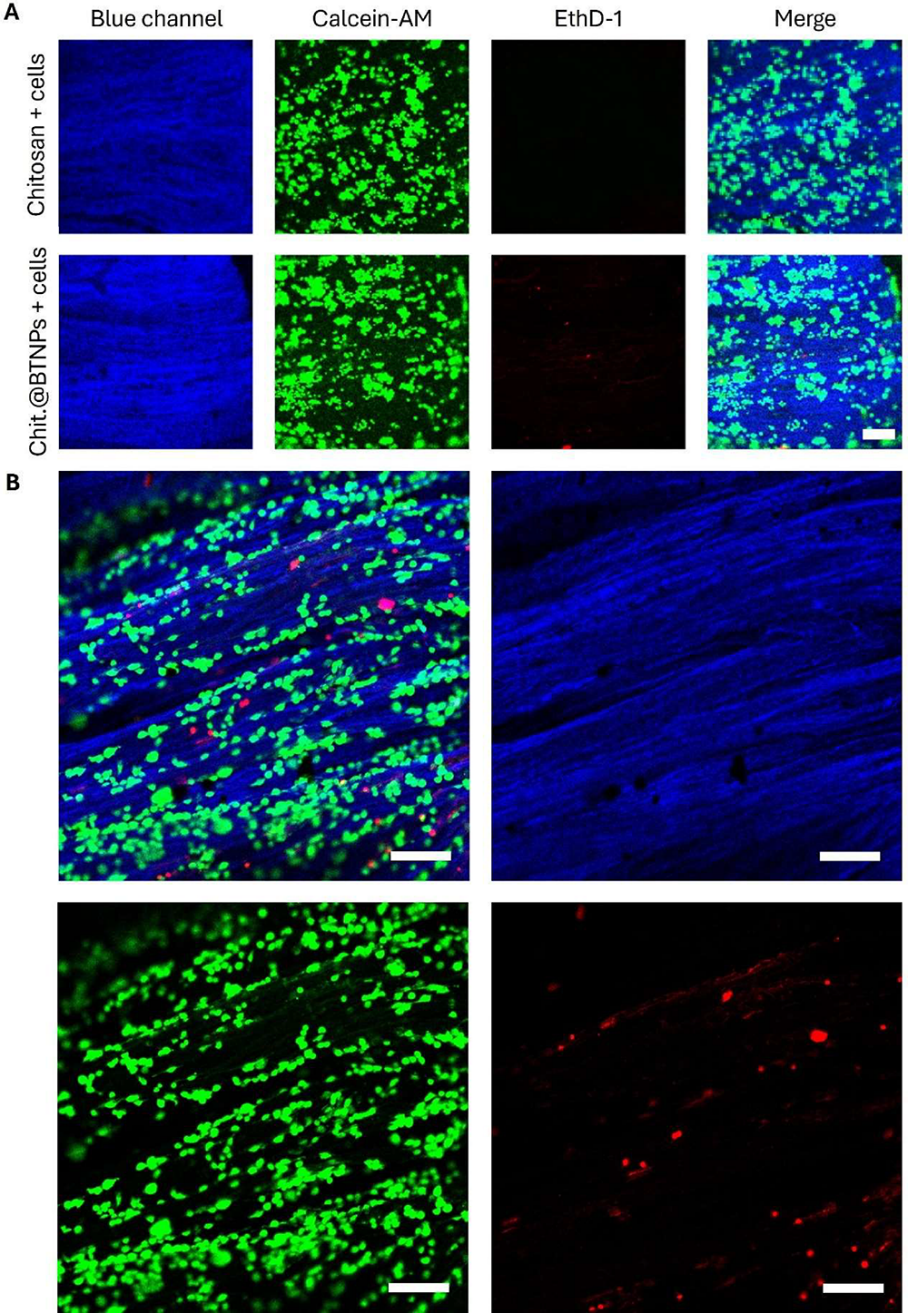
(A) Representative LIVE/DEAD images (blue: chitosan matrix; green: viable cells; red: necrotic or dead cells; scale bar 500 μm) of cell viability assay. *N* = 4. (B) Magnification of an area of the scaffold showing cell alignment along pores’ direction (blue: chitosan matrix; green: viable cells; red: necrotic or dead cells; scale bar 100 μm).

These images allow visualization of the scaffold’s porous morphology (Figure 6A, B, blue channel), enabled by laser scattering during confocal microscopy. The results qualitatively confirm unaltered SCs viability and the scaffolds’ ability to support cellular adhesion. Notably, the imaging of SCs-seeded scaffolds shows cells assembling into small clusters that align following the anisotropic pore structure, emphasizing the role of scaffold morphology in guiding cellular adhesion (Figure 6B).

These samples were then subjected to LIPUS stimulation. The overall results obtained for the different I_SAPA_ conditions explored are reported in Figure 7. Figure 7A summarizes the experimental setup of the migration assay. The scratch assay demonstrated that the empty area of Chit.@BTNPs was populated by SCs statistically significantly compared to the plain chitosan scaffold when the sample was exposed to *I*_SAPA_ of 500 and 1000 mW cm^–2^. To quantify this effect, the percentage of green pixels in the empty area was calculated, and a statistical comparison between the experimental groups was made. As shown in Figure 7B, for the groups stimulated at 500 and 1000 mW cm^–2^, a statistically significant difference was observed between plain chitosan and Chit.@BTNPs scaffolds (an increase from 8.5 to 23.4% and from 15.2 to 22.8%, respectively, considering average values). Given that both the *I*_SAPA_ of 500 and 1000 mW cm^–2^ induced a statistically significant cellular migration relative to the nonstimulated control, we selected 500 mW cm^–2^ as the optimal condition for further experiments. The rationale behind this choice is that this *I*_SAPA_ could achieve the same bioeffect on SCs while using a lower energy dose, thereby ensuring safety in future in vivo settings.

**Figure 7:**
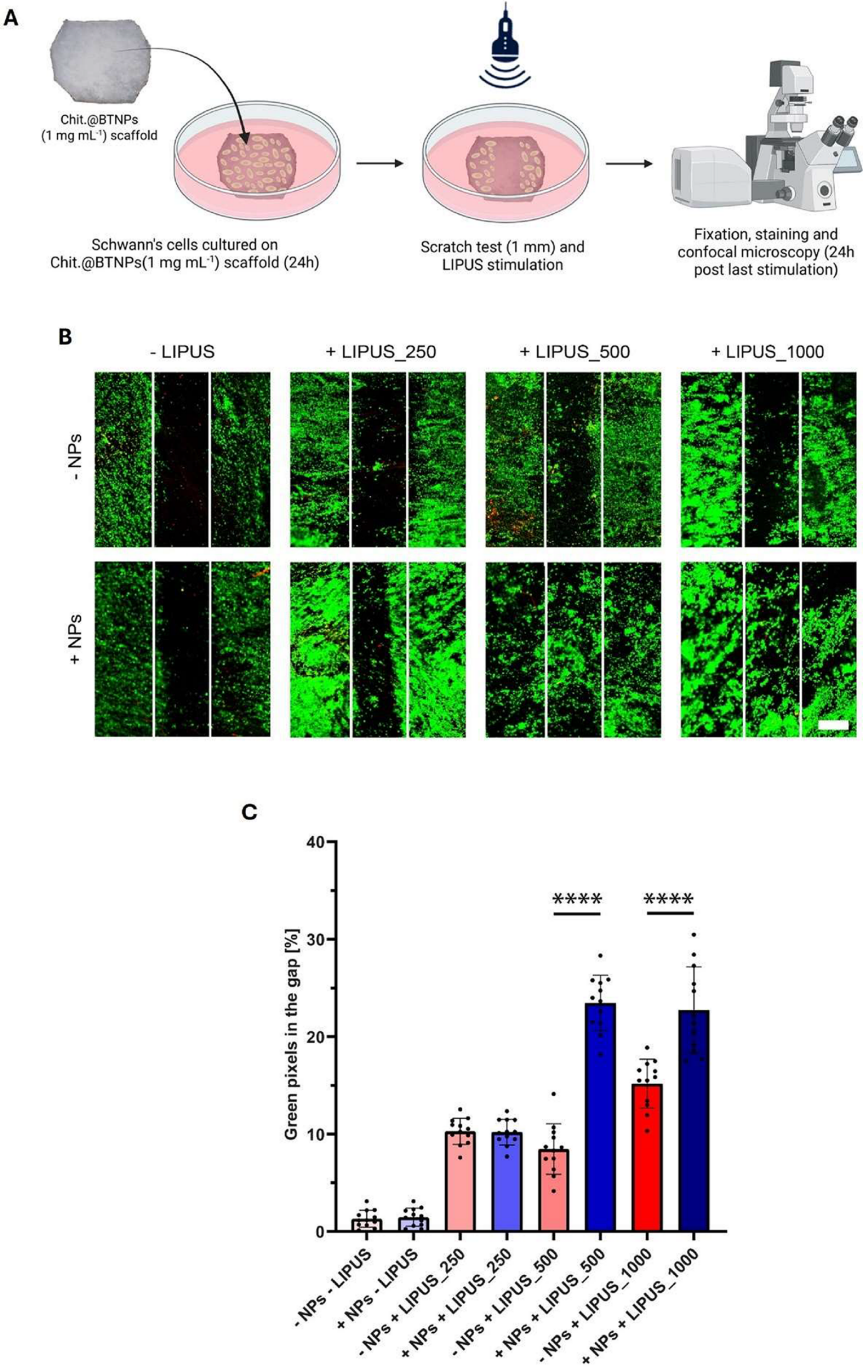
(A) Experimental setup of cell migration experiments. (B) Representative LIVE/DEAD images (green: viable cells; red: necrotic or dead cells; scale bar 500 μm) of cell migration assay, 24 h post stimulation (*f* = 5 MHz, *I*_SAPA_ = 250, 500, and 1000 mW cm^–2^, DC = 20%, pulse repetition *f* = 1 kHz, *T* = 5 min/day for three consecutive days). *N* = 4. (C) Percentage of green pixels in the gap. **** = *p* < 0.0001. *N* = 4. Created with Biorender.com.

### 3.3 SCs Metabolism and Neurotrophin Production

In Figure 8 SCs metabolism, β-NGF, BDNF, and GDNF production in samples with and without BTNPs and stimulated with LIPUS at 500 mW cm^–2^ or not stimulated are depicted. The metabolic results are reported in Figure 8A, in terms of fluorescence read at 560 nm, first normalized with respect to the dsDNA amount of the corresponding sample and subsequently normalized with respect to the average of the “- NPs – LIPUS” group. The results showed that neither the use of LIPUS nor BTNP loading was able to induce variations in cell metabolism compared to the control (“-NPs – LIPUS” group).

**Figure 8:**
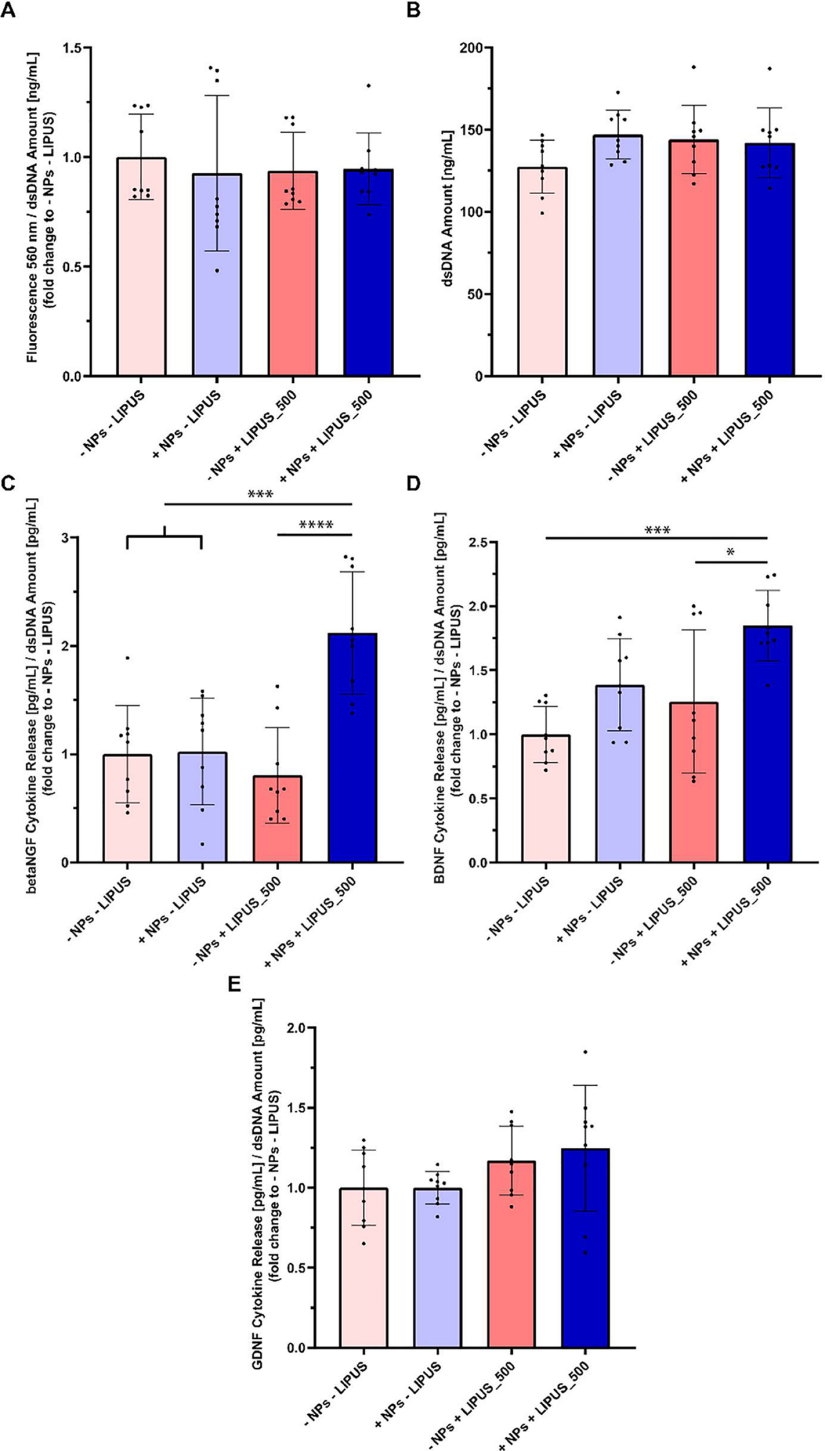
(A) Cell metabolism reported in terms of fluorescence signal read at 560 nm over the dsDNA amount, (B) PicoGreen results, represented as dsDNA amount [ng/mL]. One-way ANOVA with Tukey’s multiple comparisons test was applied (*N* = 3). (C) β-NGF, (D) BDNF, and (E) GDNF production, reported as cytokine release over the dsDNA amount. * = *p* < 0.05, *** = *p* < 0.001, **** = *p* < 0.0001. *N* = 3.

The results for β-NGF, BDNF, and GDNF cytokine release are shown in Figure 8C–E, respectively. These values are also normalized with respect to the dsDNA amount and then to the average of the “- NPs – LIPUS” group. The results in terms of the dsDNA amount, used to normalize all of the data in Figure 8, are shown in Figure 8B.

For β-NGF cytokine release, no significant differences were observed between the “- NPs - LIPUS,” “+ NPs - LIPUS”, and “- NPs + LIPUS_500” groups. In contrast, this cytokine level significantly increased in the “+ NPs + LIPUS_500” group compared with all of the others (doubling the value, considering the average of the experimental groups). For BDNF, similarly, no statistically significant differences were detected among the “- NPs - LIPUS,” “+ NPs - LIPUS”, and “- NPs + LIPUS_500” groups. However, the “+ NPs + LIPUS_500” group showed higher values compared to the “- NPs – LIPUS” and “- NPs + LIPUS_500” groups, but was statistically similar to the “+ NPs – LIPUS” group. Finally, with regard to the evaluation of GDNF release, no statistically significant differences were observed among the four experimental groups.

### 3.4 Gene Expression Analysis

A gene expression analysis was conducted to assess whether LIPUS stimulation could induce the upregulation of specific genes associated with the cellular migration processes.

We used RT2 profile PCR arrays that analyzed 84 specific genes involved in the cell motility pathway. We found an upregulation of 2 out of 84 genes in the “- NPs + LIPUS_500” group compared to the control (“- NPs – LIPUS” group, see Figure S5) and, more interestingly, 10 out of 84 in the “+ NPs + LIPUS_500” group compared to the control, while none were downregulated by the treatment (see Table 2 and Figure S5). Therefore, the gene expression analysis suggested general activation of cell motility pathways (Figure 9).

**Table 2:** Gene Expression Analysis Related to the Cell Motility Pathway^a^,^b^

gene name	fold change	p-value
CAPN1	22.85	0.0082
DIAPH1	7.47	0.0222
HGF	7.48	0.0387
ITGA4	17.50	0.0011
LIMK1	20.01	0.0039
MMP9	30.47	0.0107
PAK4	23.15	0.0425
PIK3CA	27.30	0.0106
PLD1	18.52	0.0235
PTK2B	15.61	0.0116

a Upregulated genes in the “- NPs + LIPUS_500” group, compared to the “- NPs - LIPUS” group with a Fold Change at least of ±2 and a *p*-value less than 0.05 are listed.

b CAPN1, calpain 1; DIAPH1, diaphanous homologue 1 (Drosophila); HGF, hepatocyte growth factor; ITGA4, integrin, alpha 4; LIMK1, LIM domain kinase 1; MMP9, matrix metallopeptidase 9; PAK4, P21 protein (Cdc42/Rac)-activated kinase 4; PIK3CA, phosphoinositide-3-kinase, catalytic, alpha polypeptide; PLD1, phospholipase D1; PTK2B, PTK2B protein tyrosine kinase 2 beta.

**Figure 9:**
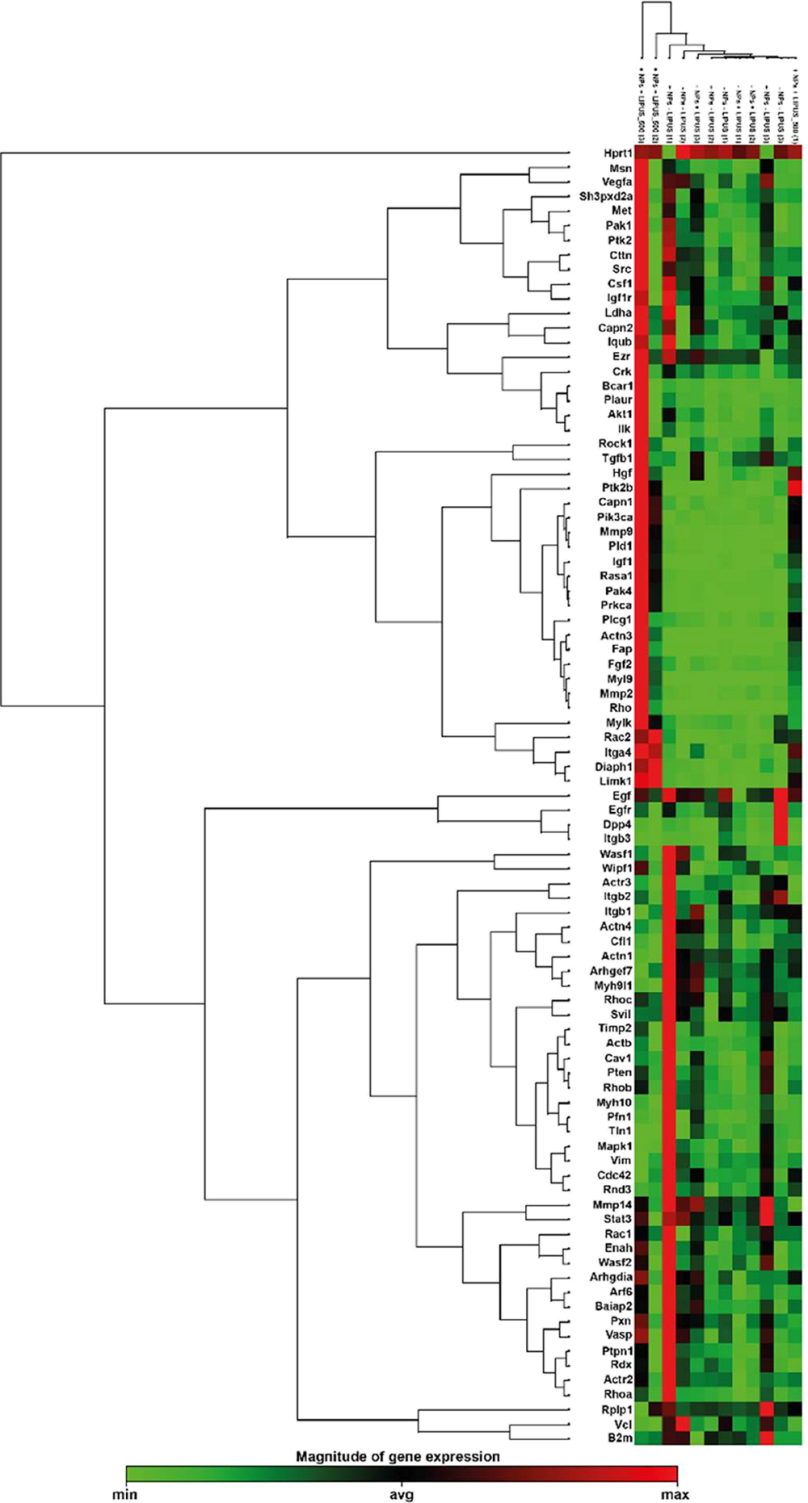
Clustergram of deregulated genes in the cell motility pathway: the cluster gram presents a heat map of the cell-motility-related genes across the selected groups. Each sample is represented three times, corresponding to biological replicates. The color scale indicates the expression level of each gene in the groups: green represents downregulation, red indicates upregulation, and black denotes no change in expression.

## 4 Discussion

Neurotmesis treatment remains a significant neurosurgical challenge, with current approaches often failing for lesions beyond the LGL, resulting in persistent motor, sensory, and autonomic deficits and refractory neuropathic pain.^47,48^ With prolonged denervation, SCs' functionality declines as their pro-regenerative phenotype gradually deactivates, resulting in poor recovery and regeneration failure.^1,49^ Numerous strategies have been explored to support nerve regeneration over the LGL.^3,50^ These include the manufacturing of topographical cues to obtain biomimetic conduits,^51,52^ and incorporating drug delivery systems for the sustained release of drugs or growth factors.^53–56^ However, these approaches have not yet received clinical approval due to a lack of regulatory clearance and their long-term instability. Conduits loaded with piezoelectric nanoparticles have been investigated in recent years,^57–59^ but showed inconsistent results, as the piezoelectric effect was triggered by animal movement, thus impeding the delivery of reproducible and quantifiable mechanical stimulation. Moreover, the literature suggests that physical exercise alone can also influence nerve regeneration,^60^ thus complicating the ability to precisely discern the relative contribution of the piezoelectric effect versus physical exercise to the observed enhancement in nerve regeneration.

An innovative approach involves the combination of LIPUS and piezoelectric materials, envisioning wireless stimulation modalities enabled by a localized generation of electrical stimuli at the cellular level in response to periodical mechanical deformation.^61^ LIPUS alone has been reported to enhance SC migration and viability in vitro.^62–64^ LIPUS and BTNPs have proven effective in regenerating different tissues, among which are cartilage^65^ and skeletal muscle.^66^

Although some studies have investigated the role of the US-activated piezoelectric effect in nerve regeneration, to the best of our knowledge, no in vitro investigations have specifically examined the synergistic interaction between LIPUS and BTNP-based piezoelectric porous scaffolds on SCs, particularly in terms of cellular functionality and gene upregulation. Although we did not directly measure the piezoelectric potential generated by BTNPs under LIPUS stimulation, our interpretation is supported by both the biological outcomes observed in this study and by prior modeling work in the literature.^21^ Direct in situ quantification of the electrical output from individual nanoparticles embedded within a 3D scaffold remains technologically very challenging. To address this, several groups have instead employed analytical and finite element models to predict the piezoelectric response of barium titanate nanoparticles under ultrasonic excitation. For instance, Marino et al.^23^ demonstrated analytically that the generated voltage scales with particle radius and ultrasound pressure, while Ricotti et al.^65^ estimated by FEM that a 60 nm BTNP can reach potentials of up to ∼43 μV at a peak-to-peak pressure of 172 kPa (corresponding to ∼250 mW cm^–^^2^). Extrapolating from these models, the intensities used in our experiments (500 mW cm^–^^2^) would be expected to generate voltages on the order of ∼60 μV. Z-potential measurements (Figure S2) confirmed the colloidal stability of BTNPs in chitosan, supporting their homogeneous distribution within the scaffold. Based on SC dimensions (∼50 μm) and BTNP localization beneath the polymeric lamellae, we estimate ∼10^5^ particles per cell. Given a piezoelectric output of ∼60 μV per BTNP, the resulting cumulative potential under LIPUS stimulation is sufficient to trigger downstream effects in electrically responsive cells, such as SCs.

A key aspect of our study is also to explore how variations in critical LIPUS parameters influence SCs' behavior in vitro, which is essential for identifying optimal in vivo settings to minimize adverse effects on surrounding tissues and maximize therapeutic efficacy.

A chitosan scaffold with an anisotropic pore microstructure was manufactured to mimic the native connective nerve architecture (Figure 3). BTNPs were successfully incorporated within the chitosan microstructure, with no significant changes in pore size, axis ratio, or angular orientation upon varying nanoceramic concentrations. (Figure 4). These results confirmed that BTNP loading did not compromise the anisotropic structure of the scaffold, whose microporous geometric architecture is compatible with cellular adhesion and proliferation processes.^67^ Such pore morphology has also been proven to provide structural guidance for axonal growth and reduction of axon misdirection.^52,68^ A noteworthy distinguishing feature of our study lies in the choice of highly cytocompatible materials^69,70^ engineered to adopt a biomimetic architecture that closely mimics native tissue structure. This design strategy significantly contributes to the overall biocompatibility of the scaffold and has been recently shown to promote nerve regeneration in vivo.^39,71^ Moreover, the mechanical characterization of the scaffolds demonstrated a linear elastic behavior under tension, with Young’s moduli of approximately 0.5 MPa for all sample groups (Figure 5), in line with previous reports on native nerve tissue.^44,46^ Preliminary in vitro viability tests showed that Chit.@BTNPs scaffolds did not cause cytotoxic effects on SCs and provided further evidence that their anisotropic porosity is beneficial for cell adhesion, as SCs tend to align according to the angular orientation of the pores (Figure 6). Furthermore, we investigated the effects of combining LIPUS and BTNPs on scaffolds seeded with SCs by sweeping several *I*_SAPA_ at a specific wavelength of 5 MHz. Our results showed that SCs migration was remarkably enhanced by the application of LIPUS on Chit.@BTNPs scaffolds compared to the control (“- NPs – LIPUS”) (Figure 7). LIPUS alone stimulated SCs migration on the plain chitosan scaffold as well, in line with previous literature reports.^63^ It is worth noting that the combination of LIPUS and BTNPs resulted in the highest cell migration compared to the control and LIPUS alone (Figure 7B,C; **** = *p* < 0.0001 for “+ NPs + LIPUS_250” vs “ - NPs + LIPUS_250” and “+ NPs + LIPUS_500” vs “- NPs + LIPUS_500”), highlighting the strong effectiveness of piezoelectric stimulation on SCs' motility. Interestingly, a modest migratory response was also observed in chitosan-only scaffolds, which can be explained by the intrinsic piezoelectricity of chitosan, already described in the literature.^72^ This baseline effect is consistent with the modest enhancement of SC motility observed here, while the addition of BTNPs markedly amplified the response under LIPUS stimulation.

It is important to point out that our evaluation of LIPUS parameters enabled the identification of a protocol that not only induces notable cellular responses but also adheres to recognized LIPUS safety standards. *I*_SAPA_ below 3000 mW cm^–2^ are classified as safe, as they do not produce detrimental thermal effects on tissues, such as inflammation or oxidative stress.^26,73^ Additionally, the *f* applied in this study aligns with standard LIPUS settings and is considered safe, as it exceeds the 0.25–0.5 MHz range previously associated with peripheral nerve damage. Although most therapeutic ultrasound applications employ 1–3 MHz, the choice of 5 MHz falls within the medical ultrasound range and provides improved resolution for superficial targets. The applied intensity (500 mW cm^–^^2^) is well below the 3000 mW cm^–^^2^ safety limit set by IEC/EN standards,^74^ and considering the typical soft tissue attenuation coefficient (∼0.5 dB/cm/MHz), the energy loss at 1–2 cm depth remains moderate, ensuring both safety and effective stimulation. Exposure to such frequencies, with pressures below 5 MPa, has been shown to induce axonal degeneration, myelin debris accumulation, and edema.^75^ Moreover, at a constant intensity, the use of higher frequencies—as adopted in this study— also reduces the risk of potentially harmful mechanical phenomena such as cavitation. Further in vitro analyses were carried out to investigate whether the combination of LIPUS and BTNPs could affect cell metabolism and the production of neurotrophic factors. Interestingly, the cellular metabolism remained unchanged across all experimental conditions (Figure 8A). Importantly, our proliferation and metabolic assays further support this interpretation. PicoGreen measurements (Figure 8B), widely employed in the literature as indicators of cell number,^76^ did not reveal any increase in proliferation across groups. These results were corroborated by PrestoBlue assays, which showed unchanged metabolic activity upon LIPUS-assisted piezoelectric stimulation. Since proliferating cells typically require increased mitochondrial activity, the stability of PrestoBlue signals further supports the absence of proliferation. Taken together, these data indicate that the biological effect of our stimulation protocol is to promote Schwann cell migration without altering their proliferative behavior. This distinction is critical as it reinforces the relevance of our findings to long-gap nerve regeneration, where effective cell migration, rather than cell division, represents the limiting factor for functional recovery. Regarding neurotrophic factor production, while this stimulation protocol did not affect GDNF release (Figure 8E), it strongly influenced not only β-NGF (Figure 8C) but also boosted the release of BDNF (Figure 8D) significantly compared to the “- NPs – LIPUS” and the “- NPs + LIPUS_500” groups. Remarkably, statistically significant differences were also reported when applying LIPUS stimulation onto a BTNPs-loaded scaffold compared to plain chitosan scaffolds (**** = *p* < 0.0001 in “+ NPs + LIPUS_500” vs “- NPs + LIPUS_500” for β-NGF in Figure 8C and * = *p* < 0.05 in “+ NPs + LIPUS_500” vs “- NPs + LIPUS_500” for BDNF in Figure 8D), further supporting the considerable impact of piezoelectric stimulation compared to the application of LIPUS alone. The differential modulation of neurotrophic factors observed in our study is consistent with their distinct biological regulation. NGF secretion is highly sensitive to mechanotransductive cues,^77^ which explains its robust increase upon LIPUS-assisted piezoelectric stimulation. By contrast, BDNF production is strongly influenced by neuron–Schwann cell crosstalk,^78^ which is absent in our monoculture system, while GDNF release is typically associated with injury-related signals and was therefore not markedly modulated under the present conditions.^79^ These results suggest that piezoelectric stimulation selectively enhances NGF production, whereas additional regulatory factors may be required to elicit significant changes in the levels of BDNF and GDNF secretion. This observation reinforces the value of future in vivo studies, where neurotrophin regulation will occur in a more complete cellular environment.

Therefore, our results demonstrated that finely tuned US stimulation increases neurotrophic factor production by SCs, consistent with previous reports where mechanical stimulation with LIPUS was applied.^35,80^ Recently, SCs have also been demonstrated through the development of complex in vitro systems as a supportive action for nerve neovascularization, acting as both physical and chemical guides for the formation of vascular channels.^81^ It is therefore reasonable to expect that the piezoelectric stimulation shown in our study may facilitate the role of SCs in the formation of neurovascular units during nerve regeneration.

To corroborate our findings, we also aimed to investigate whether our specific treatments could modulate the expression of specific genes associated with cell motility. Cellular movement is essential for various biological processes, including development and responses to infection or injury.^82,83^ Stimuli, like the release of growth factors, trigger the migration of specific cells, which involves the reorganization of the actin cytoskeleton.^84^ Regulated by the Rho small GTPase family, the actin cytoskeleton initially forms cellular projections that facilitate either the forward movement of the cell or the development of more mature structures, such as axons.^85^ Our analysis of gene expression pathways, 10 different genes were activated (Table 2, Figures 9 and S5). In particular, HGF is a well-established growth factor that promotes SCs' proliferation, migration, and survival during nerve injury and regeneration.^86^ Using LIPUS in combination with nanoparticles could enhance the release of HGF, thereby boosting SCs' activity and accelerating nerve repair. LIMK1 is involved in actin filament dynamics and is critical for SCs migration and axonal guidance.^87^ This kinase regulates the reorganization of actin filaments, a crucial process for SCs to extend their cellular projections and migrate during nerve repair. These results suggest that LIPUS-assisted piezoelectric treatment enhances SCs' motility by promoting the extension of cellular projections and remodeling of the cytoskeleton. MMP9 plays a significant role in the degradation of the ECM, enabling SCs to migrate and repair the damaged nerve.^88^ The activity of MMP9 is tightly regulated during the regeneration process, and it is known that electrical stimulation can upregulate MMP expression in Schwann cells.^89^ This gene’s responsiveness to mechanical stimuli could further explain the enhanced regenerative potential observed under US treatment conditions. PAK4 and PLD1 are key regulators of the actin cytoskeleton and cell motility, making them relevant for SCs migration during nerve repair.^90,91^ Although direct evidence linking these factors to electrical stimuli is limited, they may be indirectly activated by LIPUS, supporting SCs migration and myelin sheath regeneration. PTK2B regulates focal adhesion dynamics and cell motility.^92^ Electrical stimulation has been shown to modulate focal adhesion dynamics in various cell types, including SCs. By influencing PTK2B activity, LIPUS could enhance SCs' adhesion and migration, facilitating their movement toward sites of nerve injury and promoting effective repair and regeneration.

Overall, our gene expression analysis offers a comprehensive understanding of how the combination of LIPUS and BTNPs regulates gene expression, uncovering the underlying molecular mechanisms of SCs migration and neurotrophic factor production. Remarkably, it not only validates the in vitro evidence but also clarifies the genetic foundations of cellular functions that are critical for nerve regeneration. Ultimately, we view this study as a pivotal step in the preclinical validation of the proposed wireless stimulation technology, establishing a robust biomolecular framework to support its future in vivo validation and clinical translation in the context of peripheral nerve regeneration.

## 5 Conclusions

This study demonstrated the potential of a microporous piezoelectric chitosan scaffold loaded with barium titanate nanoparticles (Chit.@BTNPs) activated by US waves as a novel approach to support peripheral nerve repair. The scaffold demonstrated the ability to promote SCs migration and enhance neurotrophic factor production, such as β-NGF and BDNF, underscoring its potential to activate specific cellular functions upon US stimulation. Gene expression analysis further confirmed the relevance of our approach, highlighting the upregulation of pathways associated with cell motility and regeneration, and validating the efficacy of the Chit.@BTNPs scaffold in supporting nerve regeneration. By combining the advantages of piezoelectric materials and US stimulation, this approach offers a noninvasive and effective strategy to overcome the shortcomings of traditional treatments. These findings pave the way for further investigations and optimizations aimed at translating this technology into clinical applications, providing a significant step toward more effective treatments for peripheral nerve injuries.

## Supplementary Material



## Data Availability

The data that support the findings of this study are available from the corresponding author upon reasonable request.

## Notes

Figure S1: Illustration of the analysis process for the morphological characterization of the samples; Figure S2: Zeta Potential Analysis of BTNPs water-(CH_3_COOH 2% (v/v)) dispersions; Figure S3: Energy dispersive X-ray (EDX) analysis of Chitosan/BTNPs scaffolds; Figure S4: Average stress–strain curves of Chitosan/BTNPs scaffolds; and Figure S5: Gene expression analysis of genes related to the cell motility pathway (PDF)
