# Supplementary material for: Piezoelectric Chitosan Microporous Scaffolds for Ultrasound-Driven Schwann Cell Migration and Enhanced Neurotrophins Production
Source: ACS Biomater Sci Eng. 2025 Oct 14;12(1):461–75. doi: 10.1021/acsbiomaterials.5c01086 (PMC12807355; doi:10.1021/acsbiomaterials.5c01086)
Supplement: Supplementary file 1 [file ab5c01086_si_001.pdf]

Supporting Information

Piezoelectric chitosan microporous scaffolds for ultrasound-driven Schwann cell migration and enhanced neurotrophin production

Marta Bianchini[#], Francesco Iacoponi[#], Matteo Battaglini, Gianni Ciofani, Silvestro Micera, Leonardo Ricotti, Eugenio Redolfi Riva[‡,], Andrea Cafarelli[‡]*

Marta Bianchini, Francesco Iacoponi, Silvestro Micera, Leonardo Ricotti, Eugenio Redolfi Riva, Andrea Cafarelli
The BioRobotics Institute, Scuola Superiore Sant'Anna, Piazza Martiri della Libertà 33, 56127, Pisa, Italy
Department of Excellence in Robotics & AI, Scuola Superiore Sant'Anna, Piazza Martiri della Libertà 33, 56127, Pisa, Italy
E-mail: eugenio.redolfiriva@santannapisa.it

Matteo Battaglini, Gianni Ciofani
Istituto Italiano di Tecnologia, Smart Bio-Interfaces, Viale Rinaldo Piaggio 34, 56025 Pontedera, Italy

Silvestro Micera
Bertarelli Foundation Chair in Translational Neuroengineering, Centre for Neuroprosthetics and Institute of Bioengineering, School of Engineering, École Polytechnique Fédérale de Lausanne (EPFL), Lausanne 1007, Switzerland

Keywords: Schwann cells, low-intensity pulsed ultrasound, piezoelectric nanoparticles, chitosan, nerve regeneration, cell migration

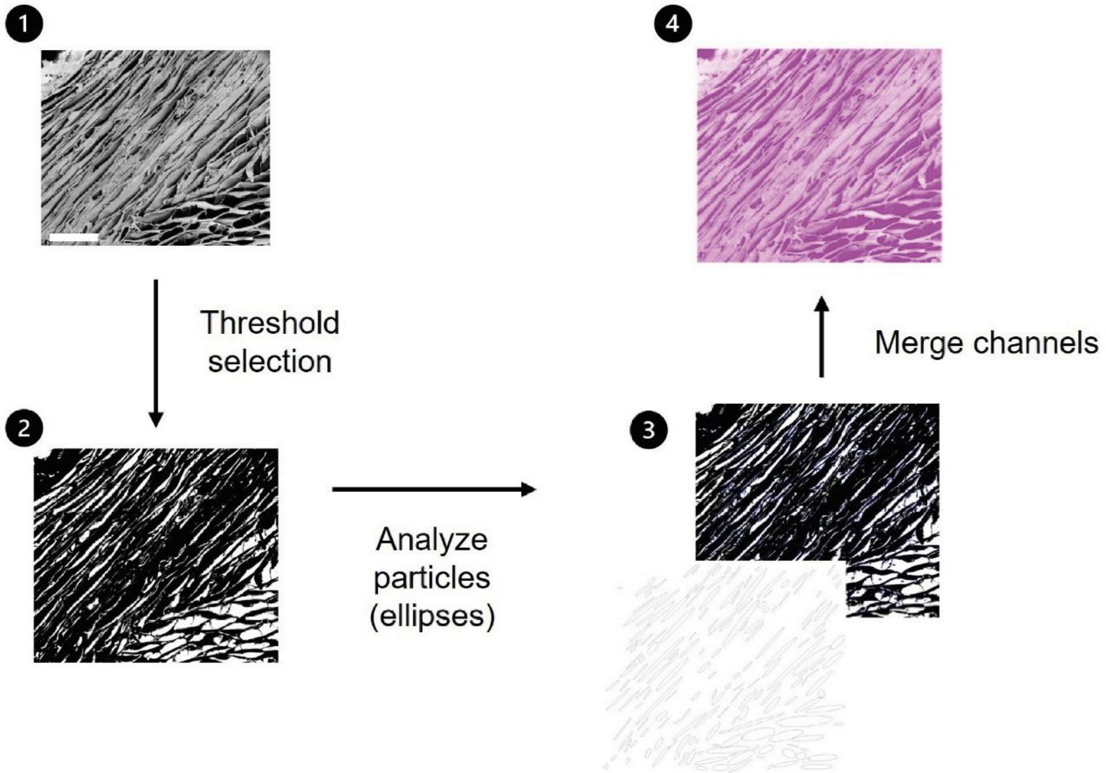

Figure S1. Illustration of the analysis process for the morphological characterization of the samples. **1)** Loading the image and setting the scale bar. **2)** Selecting the threshold to obtain a binary image. **3)** "Analyze particles" command to draw ellipses and calculate their parameters. **4)** Merging channels between the original image and the image with ellipses. Scale bar: 300 μm.

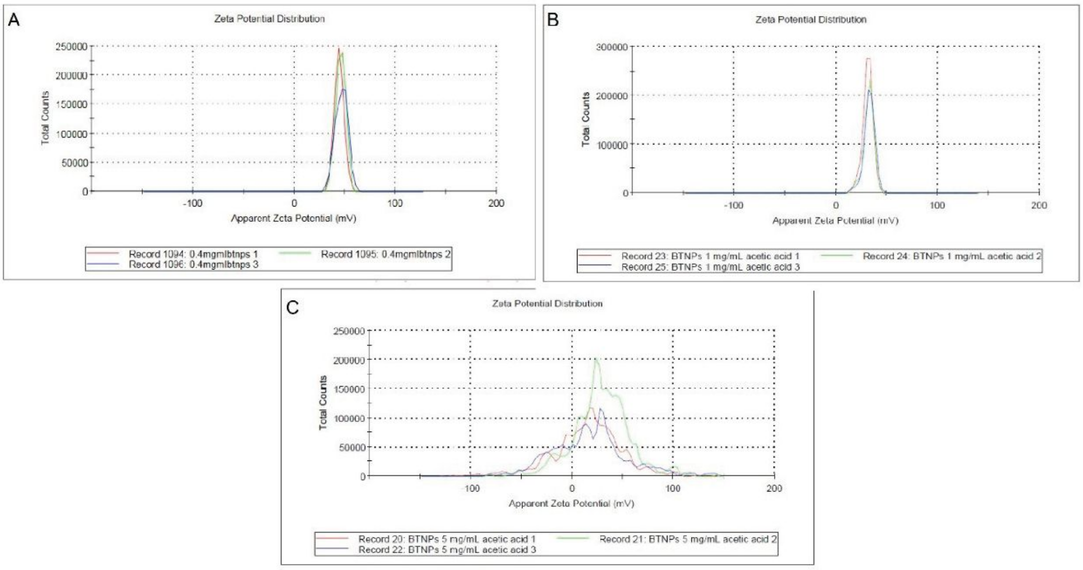

Figure S2. A) Zeta potential of BTNPs with a concentration of 0.4 mg mL^{-1} dispersed in distilled water and acetic acid 2% (v/v). **B)** Zeta potential of BTNPs with a concentration of 1 mg mL^{-1} dispersed in distilled water and acetic acid 2% (v/v). **C)** Zeta potential of BTNPs with a concentration of 5 mg mL^{-1} dispersed in distilled water and acetic acid 2% (v/v).

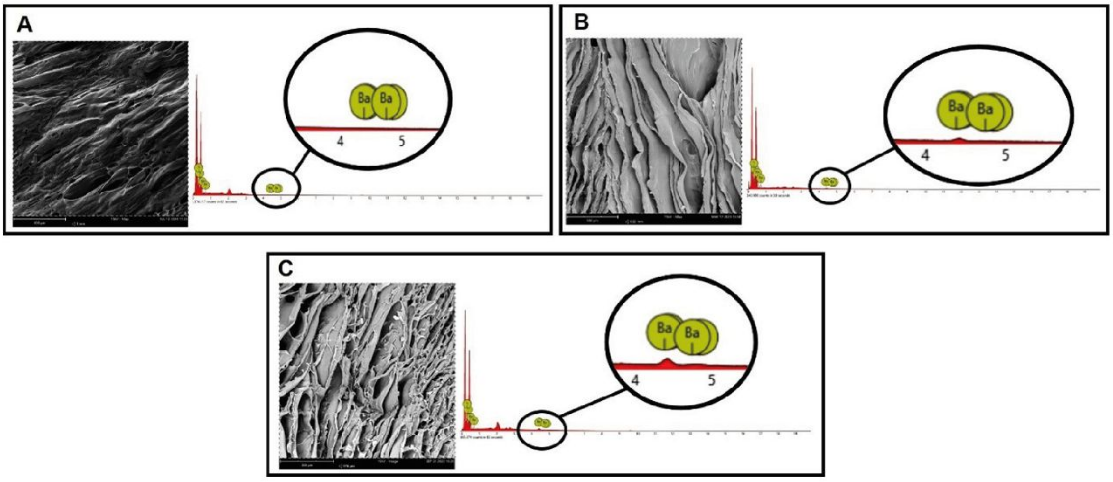

Figure S3. Energy dispersive X-ray (EDX) analysis. For each sample SEM image, EDX spectrum and magnification of the EDX spectrum in the range of 4-5 kV were reported; **A)**

EDX of plain chitosan scaffold; **B)** EDX of Chit.@BTNPs (0.4 mg mL^{-1}) scaffold; **C)** EDX of Chit.@BTNPs (1 mg mL^{-1}) scaffold.

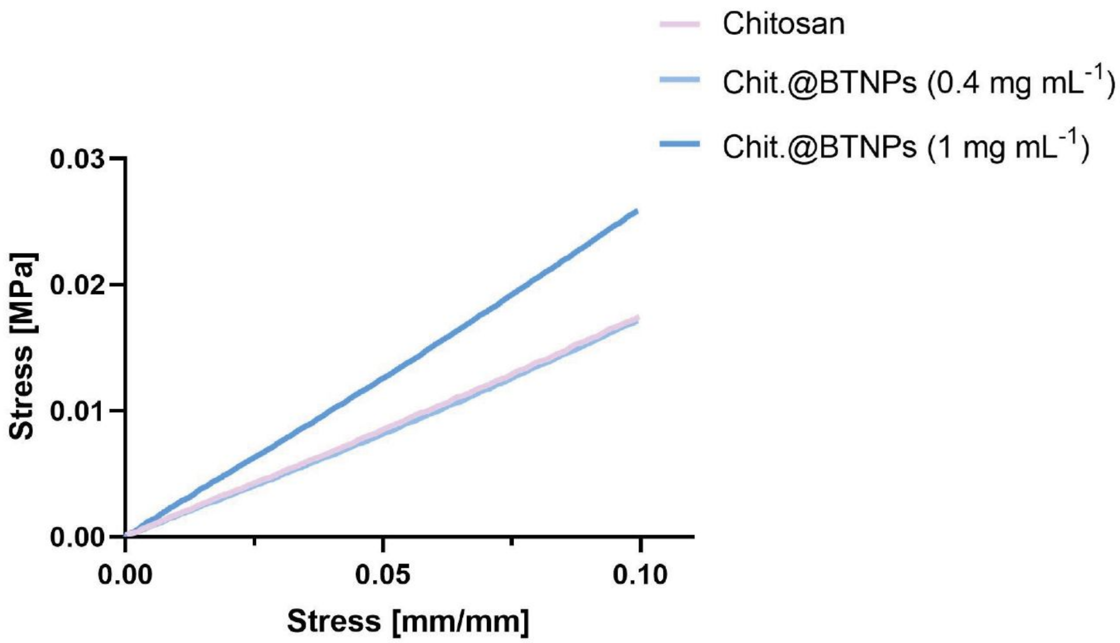

Figure S4. Average stress-strain curves of plain Chitosan, Chit.@BTNPs (0.4 mg mL^{-1}) and Chit.@BTNPs (1 mg mL^{-1}) scaffolds. N = 5.

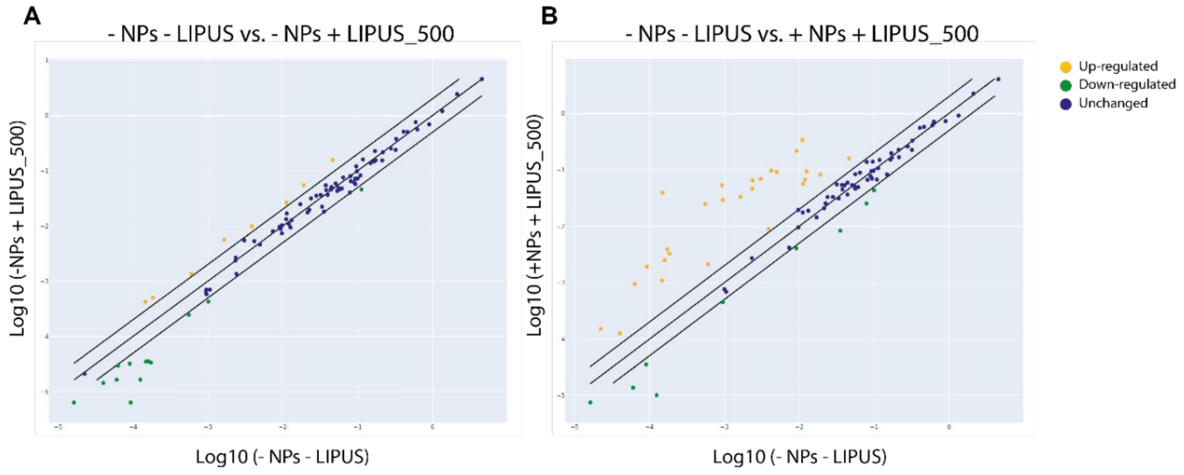

Figure S5. Gene expression analysis of genes related to the cell motility pathway. Scatter plots of MAPK-regulated genes, comparing the **A)** - NPs + LIPUS_500 and **B)** + NPs + LIPUS_500 group with respect to the control group: these plots compare the normalized expression levels of each gene in the PCR array between the two chosen groups. They visually highlight significant gene expression variations by plotting the data points, with the central diagonal line representing genes with no change in expression. The outer diagonal lines indicate the predefined fold regulation threshold. Genes that fall outside these outer lines (in the upper left or lower right corners) show significant upregulation or downregulation, respectively, beyond the fold change threshold for the y-axis group relative to the x-axis group. The scatter plot includes all deregulated genes, even those that do not show statistically significant differences.
